# Epigenetic Alterations in Sports-Related Injuries

**DOI:** 10.3390/genes13081471

**Published:** 2022-08-17

**Authors:** Maciej Tarnowski, Patrycja Tomasiak, Marta Tkacz, Katarzyna Zgutka, Katarzyna Piotrowska

**Affiliations:** 1 Department of Physiology, Pomeranian Medical University in Szczecin, al. Powstańców Wlkp.72, 70-111 Szczecin, Poland; 2 Institute of Physical Culture Sciences, University of Szczecin, 70-453 Szczecin, Poland

**Keywords:** epigenetics, miRNA, physical activity, exercise, inflammation, injuries, trauma, TBI

## Abstract

It is a well-known fact that physical activity benefits people of all age groups. However, highly intensive training, maladaptation, improper equipment, and lack of sufficient rest lead to contusions and sports-related injuries. From the perspectives of sports professionals and those performing regular–amateur sports activities, it is important to maintain proper levels of training, without encountering frequent injuries. The bodily responses to physical stress and intensive physical activity are detected on many levels. Epigenetic modifications, including DNA methylation, histone protein methylation, acetylation, and miRNA expression occur in response to environmental changes and play fundamental roles in the regulation of cellular activities. In the current review, we summarise the available knowledge on epigenetic alterations present in tissues and organs (e.g., muscles, the brain, tendons, and bones) as a consequence of sports-related injuries. Epigenetic mechanism observations have the potential to become useful tools in sports medicine, as predictors of approaching pathophysiological alterations and injury biomarkers that have already taken place.

## 1. Introduction

Physical inactivity is responsible for approximately 6% of the global burden of disease; globally, around 28% of adults aged 18 years and over did not meet the physical activity guidelines recommended in 2016 [1]. It is estimated that more than a quarter of the world’s adult population (1.4 billion adults) are insufficiently active. Physical inactivity increases the risk of many conditions, including hypertension, coronary heart disease, stroke, diabetes, and breast and colon cancers. In contrast, physical activity is a key determinant of proper energy balance and weight control [2]. From a psychological perspective, sports have many advantages, such as reducing anxiety, stress, and depression, and increasing one’s self-esteem. Moreover, regular physical activity (PA) can help individuals maintain healthy relationships with their peers; it can also be a means for people to socialize, acquire skills, and improve their competitiveness [3]. Although global statistics show that insufficient activity increased by 5% (from 31.6% to 36.8%) in high-income countries between 2001 and 2016 [1], the notable increase in the popularity of sports and the need for fitness, as well as the expectation of success in a short period of time, make physical activity-related injuries a concern [4]. Sports-related injuries are significant burdens to healthcare, have important psychological/motivation aspects, and—in professional sports—have severe economic consequences [5,6]. Among children and young adults in developed countries, physical exercise and sports are the most commonly reported causes of injury-related emergency department visits [7,8]. The main causes of sports-related injuries are injuries associated with overuse that occur after repetitive stress and micro traumas using inappropriate equipment (or not using appropriate equipment), such as protective gear in contact sports (boxing, ice hockey, etc.), commonly leading to acute traumatic injuries [9,10]. In addition, the risk of sports injuries is high for beginners who rapidly participate in practice programs, for those who start practicing intensively after a long break, or “the weekend warriors”, i.e., amateur athletes who condense intensive weekly aerobic exercises into one or two weekend sessions [9,10,11].

Injuries have various characters, progressions, and therapies, depending on the type and location of the injury [12]. Injuries might be present in almost all tissues or organs. The most frequently injured organs and tissues are muscles, tendons/ligaments, bones, and the head. Thus, with the modern contemporary active lifestyle, increased interest in regular PA, and widespread professionalism in sports, the demand for high-end injury therapies is growing. However, the developments of such treatment methods involve thorough analyses and knowledge of the processes and mechanisms involved on a molecular biology level. The investigations into the mechanisms triggered by injuries and the molecular consequences of injuries are often neglected while more attention is paid to the management of symptoms. However, understanding these processes is becoming more important, as therapies are becoming increasingly focused on specific pathways, particularly molecules and factors. In recent decades, extensive research has been carried out in the field of cellular and molecular exercise physiology [13].

Sports and exercise researchers evaluate the adaptiveness of athletes’ responses to training as well as their performance levels using a variety of physiological, biochemical, and biomedical indicators. Since the late 1990s, it has been known that genetic backgrounds are highly important in the performance-related effects of sports training but also in the vulnerability and predisposition to injuries and recovery from injuries [14,15,16,17,18]. Research shows that there are over 200 genetic polymorphisms that affect endurance, power, and muscle performances, and may be even used in gene doping [17,18]. Sports genomics as a separate scientific discipline focuses on the organisation and functioning of the genome in elite athletes and aims to develop molecular methods that may be used for sports medical practices, personalised exercise training, nutrition prescription, and the prevention of exercise-related injuries and/or diseases [18].

Recently, a new area of molecular sciences has emerged, i.e., epigenetics. Epigenetics describes the heritable changes in gene expression that occur in the absence of changes to the DNA sequence. Research shows that there are important roles involving epigenetic modifiers that affect responses to exercise training and the predisposition to injury or disease. Nearly 20 years ago, researchers ‘showed’ epigenetic regulation involving the expression of genes highly involved in sports performances and exercise physiology, such as myocyte enhancer factor 2 (*Mef2)* [19] or slow-twitching type I myosin heavy chain (MHC) [20,21]. Today, more evidence indicates that epigenetic factors, such as DNA methylation, histone modifications, and microRNAs, are tissue-specific regulators of gene expressions that constitute key links between the genotype, phenotypic plasticity, and environment [22]. Epigenetic changes are known to express the influence of the environment on the body’s molecular responses. In the context of PA, a broad spectrum of environmental factors—the physical activity itself, nutrition, emotional challenges, or pre-existing epigenetic signatures—can then determine how an individual reacts to a certain stimulus [22]. These mechanisms provide us with new molecular insights into exercise physiology, and, in the context of sports-related injuries, we are looking for new therapeutic targets that may be applied in treatment.

**Figure 1:**
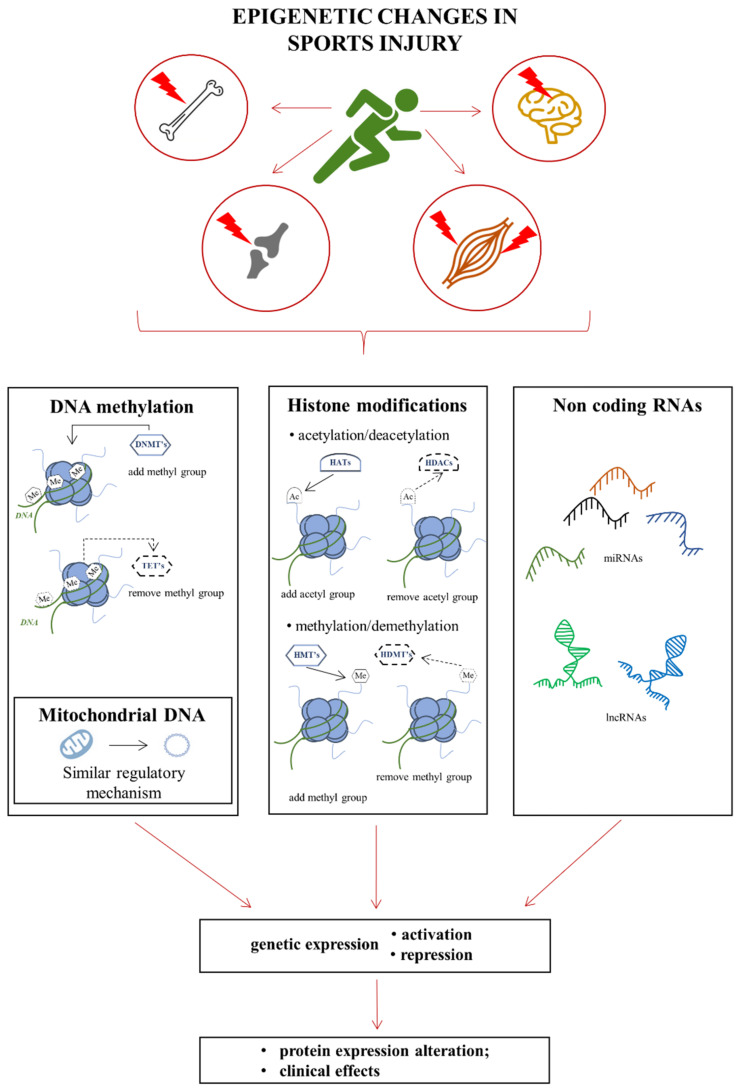
Epigenetic modifications as a result of sports-related injuries.

In this review, we focus our attention on epigenetic factors and mechanisms playing key roles in sports-related injuries, repairs, and resolutions (Figure 1). The literature search was performed in widely available databases, such as PubMed, Web of Science, and Google Scholar. We searched for articles using the following keywords and combinations thereof: epigenetics, epigenetics AND injuries, epigenetics AND inflammation, skeletal muscle injuries, skeletal muscle regeneration, brain injuries, bone injuries, tendon injuries, injuries AND miRNA, skeletal muscle injury AND sport AND miRNA. The search was completed in June 2022. Our interests include the muscles, bones, joints, the brain, and circulating epigenetic biomarkers of injuries. Thus, these six sections will constitute a review of the impact of epigenetics on injuries induced as a result of physical activity.

## 2. Epigenetics

In 2008, the scientific community agreed that epigenetics refers to developmentally or environmentally induced modifications that do not alter the DNA sequence but instead control how information encoded in genetic material is expressed in a tissue- and context-specific manner. By combining the words “epi-” and “genetics”, the term epigenetics originally suggested the idea that phenotypes arise from genotypes through programmed changes induced by the environment [23]. The term “epigenetics” was first coined in 1942 by C.H. Waddington (British embryologist) [24,25].

### Basics of Epigenetic Modifications

Shortly, in eukaryotes, chromatin is comprised of DNA segments, i.e., ~147 base pairs long-wrapped around histone octamers (H2A, H2B, H3, and H4), which constitute the nucleosome (with a single histone H1, a linker protein between particular nucleosomes). The chromatin structure can either be loosely packed into euchromatin (open chromatin) or more tightly packed into heterochromatin (closed chromatin) [26,27,28,29,30,31]. Epigenetic mechanisms, such as DNA methylation and covalent post-translational reversible modifications to histone proteins, cooperatively determine chromatin configuration and the accessibility of the DNA to the transcriptional machinery, regulating the genetic expression [32,33,34] A broad range of enzymes executes specific epigenetic modifications. The best-characterised include DNA methyltransferases (DNMTs), histone acetyltransferases (HATs), and histone deacetylases (HDACs) involved in acetylation and deacetylation, respectively, and histone methyltransferases (HMTs) and histone demethylases (HDMs) involved in methylation and demethylation of histones, respectively.

In general, histone acetylation and methylation at lysine residues forming (i.e., H3K9ac, H4K4ac, and H3K4me3, H3K36me3), are linked to transcriptional activation (permissive chromatin). Histone deacetylation and methylation at certain locations, such as trimethylation marks at H3K27 (H3K27me3) and H3K9, lead to chromatin condensation and transcriptional repression [35,36] DNA methylation is similar to adding an epigenetic tag in the form of a methyl group to a cytosine ring at carbon position 5 in the DNA sequence. This process occurs within specific DNA regions known as CpG islands, commonly found in gene promoter regions. DNA methylation is often associated with transcriptional inactivation as it prevents the binding of DNA-binding factors to transcriptional sites [37].

The classification of ncRNA molecules is based on their sizes, and thus, a distinction can be made between small noncoding RNAs (sncRNAs and miRNAs) and long non-coding RNAs (lncRNAs; >200 nt) [38]. miRNAs, which are synthesised in response to various internal or external (environmental) stimuli, can be viewed as a part of a larger genome transcription feedback loop as they target the expressions of key epigenetic enzymes, such as DNMTs, HDACs, and HATs [39,40]. On the contrary, the expressions of miRNAs are regulated by epigenetic machineries, such as DNA methylation and histone modifications [41]. Both miRNAs and lncRNAs do not directly influence chromatin architecture or DNA structures but greatly affect posttranscriptional regulation of gene expression. Mostly, these highly-conserved molecules act as negative regulators via the degradation of messenger RNAs [42,43]. More than 2500 different miRNAs have already been identified in humans [44,45]. These small, non-coding single-stranded RNAs are produced by the multistep processes of transcription, nuclear export, and cytoplasmic cleavage, and act as posttranscriptional repressors by targeting the 3′-untranslated region of mRNA. The binding of miRNA particles to the seed sequence in mRNA and the formation of the RNA-induced silencing complex (RISC) lead to its degradation and (usually) translational repression [46]. Particular miRNA may target and affect the number of mRNAs, sometimes within a single signalling pathway, creating complex, multi-level mechanisms of fine-tuning expression regulation [47,48]. lncRNAs can have a range of activities, as they are able to form RNA–protein complexes together with transcription factors [49,50]. The molecules affect RNA stability, as well as RNA splicing, translation, and degradation. Moreover, the generation of some miRNAs is also regulated by lncRNAs [49,50,51,52].

## 3. Injury to Skeletal Muscles

### 3.1. Pathophysiology of Skeletal Muscle Injury

Skeletal muscles consist mainly of post-mitotic, multinuclear muscle fibres, which make up about half the weight of the human body. Maintaining skeletal muscle functioning largely depends on the maintenance of the regenerative capacity of muscle fibres, which may be exposed to various physical and biochemical traumas causing muscle damage. Therefore, maintaining normal muscle functioning throughout life is critical to maintaining a healthy and active lifestyle [53,54]. Skeletal muscle development begins during embryogenesis, and the main cells that drive this process are myogenic progenitor cells. After birth, these cells give rise to satellite cells that, during rest, reside between the muscle fibres and the basal lamina [55]. In the case of injury, stress, or degenerative muscle diseases, satellite cells are mobilised and serve as the main sources of muscle stem cells in adults. Activated satellite cells maintain constant levels of stem cells through self-renewal, the production of new satellite cells, and committed myogenic cells that create new muscle fibres [56,57].

Exercise-induced muscle damage is the subject of intense research in the field of physical culture sciences. Muscle injuries are serious challenges for professional athletes, accounting for up to one-third of all sports-related injuries, and are responsible for a player temporarily stopping physical activity [58,59]. The greatest proportion of muscle injuries occur during sports performances and account for 10–55% of all injuries [60]. The muscles most frequently damaged during sports are the quadriceps and gastrocnemius muscles. These muscles pass through two joints and are the most susceptible to acceleration and deceleration forces [61,62]. Muscle damage is a condition characterised by transient ultrastructural myofibril disruption, loss of muscle strength, delayed muscle soreness (DOMS), limited mobility of the affected limb, oedema, systemic efflux of myocellular enzymes and proteins, or the presence of multiple aspects at the same time [63,64]. Muscle damage tends to occur through two main mechanisms: the muscle is subjected to a sudden, direct compressive force resulting in a bruise or the muscle is subjected to excessive tensile force, resulting in damage to the muscle fibres and possible rupture [65]. There are several types of muscle injury classifications, including the: duration, type, severity, or location of the injury [63]. Based on the duration of the injury, it can be classified as acute (present for less than three weeks) or chronic [66]. Another division distinguishes injuries caused by external factors (e.g., blows, falls); the most common examples are bruises and injuries caused by internal factors, which include muscle dysfunctions and overloads [67]. Due to the place/nature of the injury, both the skin and the deeper layers of the muscular tissue may be injured to different degrees [64]. Examples of such dysfunctions include the quality of muscle contractions and fatigue, which initially do not affect the structures of muscle fibres [67]. Another classification takes into account the severity of injuries and divides them into three types: mild (type I), moderate (type II), and severe (type III) [68]. Mild muscle damage does not lead to a loss of muscle functioning, as it is caused by excessive stretching of the muscle fibres, which causes pain when contracting and passive stretching. With this type of injury, mild swelling, slight tissue damage, and mild haemorrhaging may (or may not) occur. Type II, due to twisting or stretching, is associated with partial tearing of the fibres. It can cause swelling and pain in the damaged area. There may also be swelling, moderate haemorrhaging, and pain that reduces muscle functioning. Category III muscle injuries include severe sprains and strains, which often lead to complete ruptures of the muscle structures. They cause severe pain and discomfort to the injured and lead to mobility limitations. This type of injury has a much longer recovery time and requires medical intervention [63,67].

Muscle injuries are accompanied by biological changes that always follow the same order, regardless of the muscle injury type. The three main stages are destruction, repair, and reconstruction. The last two phases are closely related and overlap with each other [65]. Immediately after the injury, inflammation begins during the initial phase of destruction. A characteristic feature of this stage is the occurrence of the rupture, the necrosis of muscle fibres, and the formation of a hematoma [69]. When muscle cells are injured, a capillary rupture occurs, and inflammatory cells migrate to the site of the injury. This reaction is enhanced by the release of cytokines and interleukins from damaged myocytes and activated macrophages and fibroblasts [63,65]. The destruction phase is followed by a repair phase, during which the damaged muscle is successfully healed. This stage includes two cooperating processes: the regeneration of broken muscle fibres and the formation of a connective tissue scar [69]. Research shows that the regenerative capacities of skeletal muscles in response to injuries significantly diminish with age, and presumed to be related to the general reduction of the regenerative capacity of ageing muscle tissue [69].

### 3.2. Genetic and Epigenetic Changes Accompanying Skeletal Muscle Injuries

Exercise-induced muscle damage can be divided into the initial damage phase, which occurs during exercise, and the secondary damage phase, which is associated with a delayed inflammatory response. Eccentric stretching of myofibrils results in the disturbance of calcium homeostasis, the sarcoplasmic reticulum, and myofibrillar proteins [70]. Injuries trigger the excessive production and release of reactive oxygen species (ROS) and damage-associated molecular patterns (DAMP), which in turn have strong effects on inflammatory activators, including: TLR, NF-κB, and AP-1 receptors [71,72]. Subsequently, there is increased secretion of numerous inflammatory factors by M1 macrophages, including: inflammatory cytokines and chemokines (tumour necrosis factor (TNF)-α, interleukin (IL)-1β and IL-6, monocyte chemoattractant protein-1 (MCP-1)), prostaglandin, and substance P. These mediators promote the migration of macrophages and neutrophils to the site of damage [73,74]. In the context of sports medicine, “inflammation” includes clinical, physiological, cellular, and molecular changes to damaged tissue. In the past, inflammation caused by muscle damage was only considered an undesirable process, it has been argued that it is associated with pain and delayed regeneration [75]. However, research shows that inflammation can be an ally in the regeneration of damaged muscles [63]. Inflammatory reactions in the muscles accompany changes at the epigenetic level. Changes have been observed in the profiles of many miRNAs, including: miR-133a, miR-146a, miR-181, miR-206, miR-486, miR-378b, and miR-23a [55,56,57]. Moreover, acute physical exertion may affect the global state of methylation. Hypomethylation of the promoter regions of the *TFAM*, *PGC-1α*, *PDK4,* and *MEF2A* genes has been noted [76]. Importantly, there is high individual variability in the response to exercise-induced muscle damage, but there are more reports that certain gene variations or polymorphisms are associated with exercise-induced muscle damage. Polymorphisms of the following genes have been found in people who suffer from muscle damage as a result of exercise: *ACTN3* (R577X, rs1815739), *TNF* (-308 G > A, rs1800629), *IL6* (-174 G > C, rs180795), and *IGF2* (G > A, rs680) [77,78,79]. Understanding the causes and consequences of these genetic links to exercise-induced muscle damage may ultimately identify individuals who are at high risk of developing specific injuries.

### 3.3. Epigenetic Mechanisms of Skeletal Muscle Regeneration after Injury

The mechanical changes and metabolic stress associated with exercise-induced muscle damage stimulate different types of cells to initiate the subsequent repair and remodelling of muscle tissue. In particular, satellite cells (muscle stem cells), inflammatory cells (e.g., neutrophils, macrophages, T lymphocytes, mast cells), vascular cells (e.g., pericytes, endothelial cells), and stromal cells (e.g., fibroblasts) interact with each other in the area of the extracellular matrix of the skeletal muscle. The transcriptional regulation of muscle cell specifications has been well-characterised. Pax3 and Pax7 are among the most important regulators of myogenesis and are considered to be the main regulators of muscle cell specification and tissue formation. Both Pax3 and Pax7 are expressed in the dermomyotome, with Pax3 playing a dominant role in embryonic myogenesis, and Pax7 mainly being required in postpartum myogenesis [80]. Satellite cells can be activated by a variety of environmental stimuli, including adhesion molecules, growth factors, and cytokines released by neighbouring cells. Signal cascades, such as MAPK and insulin-like growth factor 1/protein kinase B/AKT, transmit extracellular signals to the nucleus of the muscle cell [81,82]. In response to external signals, these pathways modulate the expression of bHLH (basic Helix-Loop-Helix) transcription factors, which include muscle-specific regulatory factors (MRFs), i.e., Myf5, MyoD, Myogenin (Myog), and MRF4. MRFs cooperate with ubiquitously expressed E proteins and MEF2C (transcription regulator of myocyte enhancing factor 2) and induce the expressions of muscle-specific genes [55]. Myogenesis is controlled not only by genetic events but also by various epigenetic mechanisms, including covalent modifications of histones and transcription factors, as well as chromatin remodelling. This cooperative regulation allows for the correct timing of the full expression of the muscle-specific genetic program, controlling the fate of muscle precursors and the transition between each development phase [83,84]. The best-known epigenetic regulators of postnatal myogenesis are microRNAs. Research shows that miR-1, miR-133, miR-133b, miR-206, and miR-499 are the controllers of myogenesis during muscle regeneration and contribute to the stabilisation of neuromuscular connections [85,86]. miR-206 is highly expressed in differentiating and maturing skeletal muscles and is now recognised as a muscle-specific miRNA [87,88]. Molecular targets of miR-206 include connexin 43 (*CX43*) and histone deacetylase 4 (*HDAC4*), among others. In skeletal muscle myogenesis, HDAC4 and CX43 inhibit the expression of specific genes, and miR-206 induces the expression of specific muscle genes to inhibit CX43 and HDAC4. This process involves upregulating the expression of growth factors (e.g., IGF-I). However, TGF-β inhibits myogenic differentiation by suppressing miR-206 expression, which increases HDAC4 expression and then inhibits the expressions of specific muscle genes. Therefore, a complex negative feedback regulatory loop is formed between miR-206 and TGF-β [89]. miR-206 is also involved in the regulation of satellite cell activation during trauma. Tissue metalloproteinase 3 inhibitor (TIMP3), an endogenous inhibitor of TNF-α converting enzyme (TACE), has been shown to act as a switch-off to myogenic differentiation by regulating autocrine TNF-α release. More interestingly, miR-206 plays an important role in the regulation of TIMP3 activity, promoting myogenesis in C2C12 myoblasts [90]. It has also been shown that deletion of miR-206 in mice significantly delays muscle regeneration caused by cardiotoxin damage [90]. miR-206 can, on its own, but also in combination with other myoMiRs, positively affect the process of muscle regeneration. The exogenous application of three miRNAs, i.e., miR-133, miR-1, and miR-206 resulted in increased expressions of Pax7, MyoD, and myogenin, not only at the mRNA level but also at the protein level on days 3 and 7 after the injury, which indicates more intensive regeneration [91]. miR-181 has been found to be highly expressed during the late phase of muscle regeneration, and its action is directed by *HOX*-A11, the negative regulator of terminal differentiation [92]. Moreover, miR-1 acts as a modulator of regenerative myogenesis. A strong regulatory relationship was found between the expressions of muscle-specific miR-1 and mTOR. Research shows that the in vitro regulation of myocyte fusion, as well as in vivo muscle regeneration, occurs through the mTOR–miR-1–HDAC4–follistatin signalling pathway [93]. In a study using a mouse model of muscle damage, an increase in miR-351 was noted. It has been found that miR-351 inhibits the expression of E2f, the key regulator of the progression and proliferation of the cell cycle, and it may promote the proliferation of myogenic progenitor cells and protect them against apoptotic death in early differentiation [94]. A similar mechanism has been reported for miR-26a, which is elevated in acute muscle injury, and the knockdown of this miRNA results in delayed muscle regeneration [95].

During PA, the change in miRNA concentrations is observed, not only in muscle tissue biopsies, but large amounts of the miRNAs are further released into the circulation. They are either packed in lipid vesicles, associated with HDL, or bound to Argonaute2 and nucleoplasmin 1 proteins [96,97]. The presence of myomiRs in the circulation was, at first, associated with muscle injury and the flux of miRNAs from dead cells [96]. Further studies in marathon runners showed that levels of muscle injury biomarkers, such as creatine kinase (CK) and highly sensitive cardiac muscle troponin (hs-cTn-T), increased in a time-dependent manner, while miR expression in circulation did not [98,99,100,101]. This has led to the conclusion that there may be other mechanisms of miRNA release from myocytes [99,100]. Mooren et al. suggested that miRNAs could be produced from pre-existing premature miRNAs subsequently secreted from cells in the form of exosomes [99], which was later confirmed by Ramos et al. in mouse experiments [101]. A high-intensity run test caused an increase in miR-133a in the blood plasma of mice and, at the same time, a decrease in the miR-133a concentration in mouse muscle tissue biopsies. The authors suggested that active depletion of miR-133a affects gene expression in skeletal myocytes and represents a fast mechanism of “on/off” gene regulation [101]. However, further studies are required to confirm the active secretion of miRNAs in humans [102]. Different types of PA (aerobic/endurance performance vs. resistance exercise) cause different miRNA responses. The most important changes in levels of circulating myomiRs are presented in Table 1.

There are certain correlations between the intensity, duration of PA, and circulating levels of specific miRNAs in blood plasma [101,103]. Ramos et al. noted that miR-24 and miR-146a are responsive to intensity (speed) in a non-dose-dependent manner, but the miR-1 is dose-dependent. In the same study, the duration of the run was shown to influence the response from miR-133a and miR-222 [101]. High volume resistance exercise increases the miR-133a concentration in circulation but not in other myomiRs [97,104]. On the contrary, aerobic performances (running, cycling, rowing) affect not only miR-133a but also many other miRNAs, mostly depending on the duration of exercise. It was shown that endurance exercises, such as half-marathons, marathons, and ultramarathons (more than 50 km) cause muscle stress and damage that is reflected in the blood plasma and saliva expressions of certain biochemical markers and multiple miRNAs. Most of the studied miRs in the circulation were related to muscle tissue damage and regeneration, and upregulated during and after strenuous PA (miR-1, mir-126, miR-133a, miR-206, miR-222, miR-208b, miR-499); only three of them so far (miR-26a, miR-29b and miR-15b) have been found downregulated due to exercise [100,105]. miR133a in particular is well-documented and assumed to be an epigenetic marker of muscle injury. Its concentration in blood is now correlated with other known markers of muscle injuries and with biochemical performances and anatomical parameters in endurance runners. There have been studies showing correlations between CK and hs-cTn-T and miR-133a and miR-206 levels [99,100,107] in elite and non-elite marathon runners [99,100,107]. Mooren et al. also showed a correlation between increased miR133a and miR-206 in the circulation with VO_2max_, speed, lactate threshold, and miR133a with the thickness of the intraventricular septum [99]. Clauss et al. revealed a correlation between peak plasma levels of miR-133a and miR-1 with left atrial diameters, indicating heart remodelling due to endurance performances [100].

Adaptations to PA found in skeletal and cardiac muscles require the adaptation of blood vessels. Repeated strenuous PA causes hypertrophy of muscles followed by remodelling of vessels and “de novo” angiogenesis. The angiogenesis process is regulated by specific miRs, such as miR-26, miR-125a-5p, miR-126, and miR-222. miR-126 is found in endothelial cells and its presence in the general circulation may indicate endothelial damage as a result of exercise.

Uhleman and co-workers showed that damage to the vessel wall and the release of miR-126 to plasma begins 30 min from the beginning of submaximal endurance PA (bicycling) and is found in other endurance tests, i.e., the maximal spiroergonomic test and marathon running [104]. Only in the case of marathon running is damage to the endothelium present together with damage to the muscles, as indicated by the presence of miR-133a in the circulation [104]. In the spiroergometry test and the four-hour bicycle test, miR-133a was unchanged vs. baseline values. In the same study, in resistance training, levels of miR-126 were unchanged, but miR-133a was elevated, suggesting damage to muscles but not blood vessels by an eccentric load [104]. Levels of miR-126 remained elevated for 1 h post-exercise and returned to baseline levels within 24 h [104,108]. The relation between PA endurance and circulatory miR-126 was confirmed in ultramarathon races. Angiogenic miR-125a-5p and miR-126 were elevated in plasma after a 100 km run [105]. Expression of miR-222, similar to miR-126, was connected to endurance exercise volume and responses for increasing exercise duration [101,106]. “Angiogenic” miR-26 was also found to be increased in extracellular vesicles present in sweat after cycling training, but miR-26, miR-126, and miR-222 were also found in the sweat of non-training subjects [109].

The other sources of circulating miR-125 and miR-126 after exercise are peripheral blood mononuclear cells (PBMNCs). Radom-Azik et al., in a series of studies with single-bout interval cycling training, showed changes in the miRNA signatures of PBMNCs [110,111,112]. In neutrophils, the expressions of 38 miRNAs were altered, in addition to 23 miRNAs in NK cells and 34 miRNAs in lymphocytes and monocytes [110,111,112]. Most of them are important in immune responses, cancer, and angiogenesis [112,113,114].

The role of other epigenetic modifications in post-injury muscle repair is not yet fully understood. Histone acetyltransferase p300/CBP and PCAF have been implicated in the regulation of muscle-specific genes [113,114]. The ability of MRF to activate proliferating myoblast differentiation is countered by the association of muscle regulatory regions with histone deacetylases (HDACs) and corepressor complexes, including Yin Yang 1 (YY1) and polycomb proteins that prevent the premature expression of muscle genes by promoting histone modification [115]. Chromatin remodelling is related to the activation and differentiation of satellite cells. Stem cell pluripotency is due to the permissive chromatin state, which is characterised by the general absence of repressive lysine 27 trimethylation on histone H3 (H3K27me3), and the concomitant presence of permissive lysine 4 trimethylation on histone H3 (H3K4me3). For example, Pax7 expression changes its chromatin state from transcriptionally active (increased H3K4me3 level) to a repressed state (increased H3K27me3 level) with an advancement in the degree of satellite cell differentiation [116]. In another study, HDAC4 was found to be critical for skeletal muscle recovery after injury, with expression peaking in the early stages of recovery. HDAC4 knock-out mice are less able to regenerate skeletal muscles after injuries. HDAC4 not only promotes the renewal and differentiation of satellite cells in an autonomous manner but also influences the proliferation and differentiation of muscle-derived cells through muscle-derived soluble factors (including FGF-1 and TGF-β) [117]. Long non-coding RNA may also be involved in the regeneration process of damaged muscles. In vivo tests have revealed that Malat1, H19, linc-MD1, linc-YY1, Sirt1 AS, and lnc-mg can modulate myogenesis and muscle regeneration [118,119,120]. Moreover, lncRNAs have been shown to positively correlate with MyoD and/or myogenin levels, and a positive correlation was observed between linc-MD1, Sirt AS, and H19). It is also believed that the regeneration of blood vessels in injured skeletal muscles is modulated by the interaction of Malat1 with HIF-1α and Angpt1 [121].

## 4. Injury to Tendons

### 4.1. Pathophysiology of Trauma

Tendons connect the muscles to the bone and allow the force generated by the muscles to be transmitted to the bone, causing the joints to move. Injuries to ligaments and tendons annually affect more than 100 million people worldwide and are described as the most commonly damaged musculoskeletal structures [122,123]. The tendons, especially in the case of physical exertion, are exposed to high loads. Tendon overload causes pain and swelling in the affected tendon, as well as associated discomfort, reduction of load tolerance, and functionality during exercise [124]. Chronic exercise is likely to be responsible for tendon overload problems in 30% of all running injuries, and elbow tendinopathy in 40% of tennis players [122]. In turn, shoulder tendinopathies constitute > 30% of referrals for musculoskeletal injuries [125]. Achilles tendinopathy is especially common in running and jumping athletes [126]. Tendon injuries can be acute or chronic and are caused by internal or external factors, isolated or in combination. In acute trauma, external factors dominate, while in chronic cases, internal factors also play a significant role [127]. In many cases, the exact aetiology and pathogenesis of spontaneous tendon rupture remain unknown [128]. Commonly, degeneration of the tendon followed by a rupture is a multi-etiological disorder. It is caused by various forms of degenerative tendinopathy and is usually associated with a mixture of predisposing hereditary factors (Ehlers-Danlos, Marfan syndrome, osteogenesis imperfecta), genetic variability (microsatellites, SNPs, copy number variants), structural factors (anatomical differences), as well as professional and lifestyle-related factors (exercise, drugs, such as fluoroquinolones antibiotics and steroids, nutrition) [128,129,130]. Moreover, important factors predisposing to a tendon rupture are of a biomechanical nature, such as local connective tissue pathologies causing friction (osteophytes, spurs, excrescences), vascular damage, and hypoxia, leading to degeneration and weakening of the tendons. The anatomy of the tendon, its vascularisation, and surrounding structures may predispose to mechanical wear of the tendon, microtrauma, or macro-trauma, and then incomplete regeneration [128]. The decline in muscle strength and power progressing with age also plays an important role in the damage and rupture of the tendon [127]. It is believed that due to the loss of collagen and its cross-linking, the tendons become stiff [123,128], which makes them more susceptible to injuries. Resistance training in old age may partially reverse the effects of ageing and improve the properties and functioning of tendons [128]. In addition, the incidence of tendinopathy is increased in individuals with reduced insulin sensitivity as seen in patients/athletes with type 1 and type 2 diabetes (T1/T2DM) [131]. Other factors that increase tendon stress include training errors, such as poor technique [132], rapid acceleration/deceleration movements [133], or inadequate sports equipment [134]. Interestingly, the lack of exposure to an appropriate level of physiological stress for a long time or “underload” may paradoxically predispose to overload injuries [135]. An underloaded tendon may not be able to meet the subsequently increased requirements imposed on it. Accordingly, underutilisation of the tendon can result in an imbalance between matrix metalloproteinases (MMPs) and their inhibitors (tissue matrix metalloproteinase inhibitors, TIMPs), leading to tendon degradation [131].

### 4.2. Molecular and Genetic Basis of Damage

In general, it is not well understood how genetics influence the susceptibility to tendon injuries. However, some specific genetic mutations have been found and linked to tendon injuries. A relationship has been noted between AB0 blood groups and spontaneous tendon ruptures, as well as a relationship between HLA blood groups and tendon diseases [128,130,136]. Kujala et al. compared patients with Achilles tendon rupture to the control population and found a significantly lower A:0 ratio [137]. In contrast, Achilles tendon ruptures in children have only been seen in children whose parents have experienced tendon ruptures in the past. Subtle gene defects in protein metabolism (i.e., collagen fibres) may explain some spontaneous tendon ruptures in apparently healthy adults, such as athletes [128]. Gene expression assessment in the ageing tendinopathy has confirmed increased matrix reorganisation, with a catabolic imbalance. Various studies have shown increased expression of *COL1A1* [138,139] and other proteins, such as COL2A1, aggrecan, and SOX9, typical of cartilage [138,140]. A significant relationship between the *COL11A1* rs3753841 genotype and elbow tendon pathology has been found [141]. Increased levels of various MMPs have also been observed in tendinopathy, including MMP23 [138,142], disintegrin, and metalloproteinase 12 (ADAM12) [138,142], and a decrease in MMP3 levels. On the other hand, Peffers et al. observed low expression levels for COL2A1, aggrecan, SOX9, most MMPs (except MMP3), and a significant reduction in ADAM12 in the old group [143]. Therefore, these results suggest that degeneration is not an inevitable consequence of ageing and that ageing and disease-associated degenerations are distinct processes [143]. Other studies have shown that muscle and tendon injuries in cyclical sports athletes are associated with single-nucleotide variants: SNV rs1800012, rs1107946 of the COL1A1 gene and SNV rs12722 of the COL5A1 gene, SNV rs679620 of the MMP3 gene, SNV rs2289360 of the ELN gene, SNV rs143383 of the GDF5 gene [135,144,145].

### 4.3. Epigenetic Changes as a Consequence of Damage

Tight control over gene expression is associated with regulatory mechanisms in cells, which can be either inducible or epigenetic [125]. In addition to biochemical and molecular signals and pathways, epigenetic mechanisms are involved in the initiation, progression, and regulation of inflammatory responses within injured tissues [146]. During asymptomatic tendinopathies, the tenocytes express the TREM1 molecule and function, such as immune cells, which in turn are regulated by high mobility group box 1 protein (HMGB1) and the receptor for advanced glycation end products (RAGE), which mediate sterile inflammatory responses [125]. Several miRNAs have been reported to be involved in tendon disorders and tendon inflammation [147]. The miRNAs associated with the pathogenesis of tendinopathy are responsible for the deterioration of tendon adhesion by mediating oxidative stress, inducing tenocyte apoptosis, or improving the healing of tendon injuries by regulating angiogenesis and related to mechanical stimuli [147]. miRNAs found in tendon and bone injuries are shown in Table 2.

miR-499 was found to regulate two specific genes, i.e., MYB and CUGBP2. MYB is a transcriptional activator and plays an important role in the control of stem cell proliferation and differentiation [148]. CUGBP2 is an RNA-binding protein implicated in the regulation of several post-transcriptional events involved in pre-mRNA alternative splicing, as well as mRNA translation and stability [162]. Specific miRNAs influence different stages of tendinopathy. miR-28 and miR-17-92 mediate oxidative stress-induced tenocyte apoptosis; miR-421-5p regulates the local expression of metalloproteases (MMPs) 2 and 9 and drives angiogenesis by increasing vascular endothelial growth factor (VEGF) production [149,150]. Moreover, Millar et al. found a correlation between the expression of miR-29a and the development of tendinopathy through the regulation of IL-33 [151]. Plachel et al. observed that several miRNAs can be significantly deregulated in patients with chronic tendinopathy (Table 2). Moreover, both miR-29a and miR-29c showed progressive repression depending on the severity of the tendon pathology. By controlling the synthesis of IL-33, miR-29a positively regulates the expression of collagen 3 (COL3A1) in tenocytes. Downregulation of miR-29a overexpressing COL3A1 has resulted in an unbalanced ratio of type 1 and type 3 collagens, which are hallmarks of tendon degeneration [152]. Moreover, in the equine therapeutic model, Watts et al. showed a significant reduction in COL3A1 synthesis following the overexpression of miR-29a [152]. In turn, miR-324 inhibits the expression of MMP-2 and MMP-9 and might promote tendon disorganisation [152], while miR-30a has been shown to promote cancer cell apoptosis and can significantly inhibit cell proliferation and aberrant ECM deposition [163]. miR-140-3p negatively regulates nuclear factor-κB (NF-κB) inflammatory signalling by limiting the expression of nuclear receptor co-activator 1 (NCOA1) and nuclear receptor-interacting protein 1 (NRIP1) [153]. miR-425 repression has also been demonstrated to correlate with the upregulation of inflammatory cytokines [164]. Thankam et al. found 12 downregulated miRNAs and one upregulated miRNA (hsa-miR-297) to be associated with 216 genes in a rotator cuff tendon injury [154]; Table 2. It is also worth mentioning that miR-148a-3p promotes thrombospondin-4 expression and enhances angiogenesis during tendinopathy; miR-124 suppresses collagen formation of the human tendon [155], and miRNA124-3p directly binds and suppresses the expression of early growth response-1 (EGR1) and suppresses the synthesis of collagen during tenogenic differentiation (the transcription factor EGR1 promotes tendon repair and regulates tendon differentiation) [149]. miR-30b-5p is involved in cartilage degradation in rats with chronic exercise arthritic injury and regulates chondrocyte apoptosis and migration by targeting Hoxa1 [156]. Age-related cellular dysfunctions have been hypothesised to result in musculoskeletal age-related diseases, such as osteoarthritis, osteoporosis, and tendinopathy. Peffers et al. have shown that miRNAs may play a role in tendon homeostasis. mir-500 and miR-548 may be regulating processes associated with matrix remodelling, which is important in tendon formation and maintenance, as well as healing. Interestingly, miR-548j is predicted to target peroxisome proliferator-activated receptor γ (PPARγ), a gene differentially expressed in tenogenic tissues from young and older MSCs donors [157]. Physical activity induces changes in DNA methylation patterns and influences the expressions of many genes in multiple tissues. Mendias et al. studied specific miRNA expression patterns in an in vivo model of tendinopathy induced by overuse (a 30-min run on a treadmill) and observed increased general cell proliferation and elevated extracellular matrix gene expression [165]. In turn, Rickaby et al. showed epigenetic alterations to a member of the MMP gene family in human patellar tendinopathy (PT). They observed differences in the methylation status of a single CpG site–65 base pairs (bp) upstream of the MMP11 promoter between the PT group and controls [166]. Khoury et al. detected a significant difference in the methylation status of one CpG site, approximately 3kb upstream of the ADAMTS4 gene between the PT group and controls [167].

### 4.4. Epigenetic Mechanisms of Tendon Regeneration

**Figure 2:**
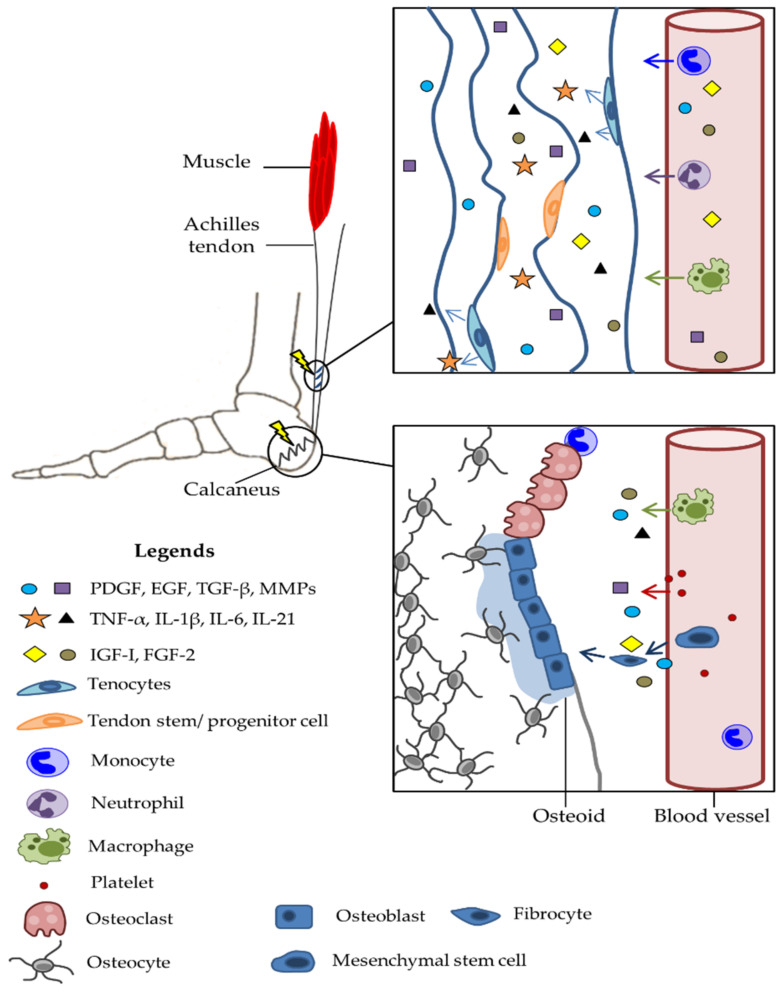
Physiology of tendon and bone regeneration.

The healing of ruptured tendons remains a clinical challenge because of its slow progress and relatively weak mechanical force at an early stage [168]. Prolonged healing of tendons occurs due to their features, such as hypovascularisation and low metabolic activity, which increase hypoxia tolerance during exercise [122]. In the first 24 h after trauma, in the inflammatory phase, erythrocytes, neutrophils, monocytes, and macrophages enter the site of injury [122]. In this process, inflammatory mediators and cytokines are secreted into the extracellular matrix [125]. Tendon injuries induce a strong chemotactic response. Released cytokines and growth factors (platelet-derived growth factor, PDGF; epidermal growth factor, EGF; transforming growth factor-β, TGF-β; MMPs) stimulate the proliferation and migration of cells to the site of damage from the tendon and synovial sheath [169]. In the tendon, collagen and α-procollagen are synthesised, and phagocytosis of collagen fragments resulting from damage is stimulated. Tenocytes under the influence of an inflammatory trigger/stimulus tend to express inflammatory cytokines, including TNF-α, IL-1β, IL-6, IL-21, and TGF)-β [125]. Tendon stem/progenitor cells (TSPCs) are types of MSCs. TSPCs support regeneration at the site of the tendon injury, and the loss of their functioning with advanced age causes aged-related tendon diseases. Compared to tenocytes, they have stem cell markers, proliferate fasters, exhibit multi-differentiation potential, and have higher expressions of tenocyte markers [170]. miRNAs also participate in tendon regeneration and repair. The miR-34 family has been shown to be pro-apoptotic via the suppression of sirtuin1 (SIRT1) and regulate the transforming growth factor (TGF)-β signalling pathway, which is necessary for TSPC maintenance and differentiation. They regulate proliferation, tendon adhesion, tendon ECM, tendon homeostasis, and promote/inhibit tenogenic differentiation [171], Table 3, Figure 2.

In turn, the miR-181 family has extensive regulatory functions in apoptosis and mitochondrial functioning while the miR-199 family regulates cell survival and proliferation [172]. Moreover, lncRNAs may limit the ability of the TSC pool to respond to the loss of differentiated tenocytes from senescence or apoptosis, reducing the functional cellular component of the ageing tendon [172]. lncRNA H19 plays an important role in tenogenic differentiation by directly suppressing the action of miRNA29b-3p, promoting activity of the TGF-β signalling pathway [173]. Peffers et al. identified alterations in 12 pseudogenes in ageing human Achilles tendons [143]. Tissue-specific methylation patterns could explain characteristic cellular phenotypes, and their relationships with cellular functions since this directly affects the transcriptome. CpG hypomethylation at CpG islands in promoter regions is linked to leprel, foxf1, mmp25, igfbp6, and peg12. In tendon constructs derived from young and old MSCs, 50% of the top 20 differentially methylated CpGs were neighbouring transcription factor encoding genes, the functions of which revealed the same expression profiles in the cellular proteins [157]. Another study identified differentially methylated CpGs associated with genes of interest in the patellar posterior, central, and anterior cuff tendons, by assessing diseased and healthy patellar tendons, the Adamts4 CpG-2995bp upstream of the promoter, and CpG +61bp upstream of the first MMP11 exon [173]. A study of histone modifications in ageing tendon tissues investigated the effects of histone methyltransferases (G9a, G9a-like protein, PR domain of zinc finger protein 2 (PRDM2), SUV39H1, SUV39H2, SETDB1/ESET) and their roles in tenocyte differentiation [174]. Another study investigated stem cell differentiation into tendon cells [175].

## 5. Bone Injuries

### 5.1. Pathophysiology of Trauma

Bone is a well-vascularised tissue, and the endothelium of the blood vessels has a critical role in the homeostasis of bone integrity [176]. Bone fractures, in most cases, are the result of trauma or specific bone diseases. Fractures can also occur as a result of a build-up of microfractures in healthy bones, called “stress fractures” [176]. Poor training techniques and various risk factors can lead to stress fractures. The basic principle of the bone response to stress is Wolff’s law, according to which, changes in stresses exerted on the bone lead to changes in its internal architecture [177]. Stress fractures, defined as microfractures of the cortical bone tissue, affect thousands of athletes annually [178]. In some disciplines, such as athletics and gymnastics, athletes show a higher rate of stress fractures [179]. An untreated stress fracture can lead to a complete bone fracture, which may require surgical anastomosis and a significant break in training [180]. The incidence of stress fractures in the general athletic population is less than 1% but may be as high as 15% in runners. Stress fractures of the tibia, metatarsals, and fibula are the most frequently reported sites [181,182]. The sites of stress fractures vary from sport to sport, i.e., among track athletes, stress fractures of the navicular, tibia, and metatarsal are common; in distance runners, it is the tibia and fibula; in dancers, the metatarsals) [181]. The occurrence of these injuries is caused by repetitive submaximal stresses on the bone. The resulting microfractures coalesce and form complete fractures. Typical treatment of low-risk stress fractures includes rest and limited weight bearing. High-stress fractures more often require surgical treatment [182].

Repeated mechanical stress results in increased activity of osteoclasts in relation to the formation of new osteoblastic bone, which in turn leads to transient bone weakening [179]. In the process of adaptation, a new periosteum is formed to provide structural strengthening [183]. Disturbance of bone density is prevalent among athletic women. The severity of bone loss ranges from osteopenia to postmenopausal osteoporosis. In female athletes, a higher incidence of osteoporosis is due to a decreased rate of bone formation in youth, caused by hormonal deficiency and/or excessive exercise. Low bone mass poses a particular challenge for athletes because it predisposes them to stress-related bone injuries and increases the risk of osteoporosis and insufficiency fractures with ageing [184]. On the other hand, in endurance runners specifically, most studies have shown a higher bone mineral density (BMD) than control populations (tibia, femoral neck, calcaneus). However, athletes from other weight-bearing sports, such as sprinters or gymnasts, have higher BMD than endurance runners. In addition, master athletes over the age of 65 years who are still competing in running events have been shown to possess higher BMD than their non-active counterparts [185].

### 5.2. Molecular and Genetic Basis of Damage

Exercise leads to bone adaptation and this process is mediated by cellular mechanotransduction [186]. Upon exercise, bone tissues deform, and the mechanosensors located throughout the cells, such as stretch-activated ion channels and integrins, change their original conformations [187]. The ability of osteocytes to detect and respond to mechanical strains leads to the control of bone formation and resorption through the differentiation of osteoblasts and osteoclasts and by stimulating the expression of the osteoclastogenesis inhibitor, osteoprotegerin [188]. Exercise activates the Wnt/β-catenin signalling pathway, which leads to osteogenesis and bone formation by direct stimulation of the bone transcription factor RUNX2 or by crosstalk with the parathyroid hormone (PTH) or bone morphogenetic protein (BMP) signalling pathways [187]. It is interesting that in athletes using one limb more actively, such as fencers or tennis and baseball players, a greater bone mass is observed in the more active limb [187]. On the other hand, limitations of muscle contractions and ground-reaction forces reduce the bone mass density of astronauts living under zero gravity conditions [187,189]. Grimes et al. evaluated differences in BMP-2/TGF-β signalling, associated with the coordination of skeletal and vascular development during endochondral ossification [190]. They found that angiogenic mRNA (VEGF, VEGF-C, CD31, and VEGFR2) and mRNA of transcriptional regulators of chondrocyte differentiation and cardiac morphogenesis (Hand2 and FoxC2) are potential transcriptional regulators of fracture healing [190].

Osteogenesis imperfecta (OI) is a group of connective tissue disorders with a broad range of phenotypes, primarily characterised by bone fragility. In most cases, there is a reduction in the production of normal type I collagen (COL1) or the synthesis of abnormal collagen as a result of mutations in COL1 genes [191]. Friedman et al. compared DNA samples from cases with high-grade SF and healthy controls and three missense mutations in the NEB, SLC6A18, and SIGLEC12 genes; three synonymous mutations in the ELFN2, GRK4, and LRRC55 genes displayed significantly different rates in SF cases compared with the control [192]. The pathway analysis showed their participation in processes, such as: cell development, morphology, survival and death, cell-to-cell signalling and interactions, the humoral immune response, the inflammatory response, nervous system development, and functioning, and tissue development [192]. In turn, Varley et al. detected that SOST SNP (rs1877632) and VDR SNPs (rs10735810 and rs731236), Wnt signalling, and vitamin D SNPs, respectively, were associated with stress fractures in a group of 518 elite athletes (football, cricket, track and field, field hockey, gymnastics, rowing, and boxing) [193]. In another study, Varley et al. reported that the variant allele of P2X7R SNP (rs3751143) was associated with stress fracture injury while the variant allele of rs1718119 was associated with reduced multiple stress fracture cases in elite athletes [194].

### 5.3. Epigenetic Changes as a Consequence of Damage to the Bone Tissue

Almost all physiological processes involved in bone remodelling are tightly regulated by epigenetic factors. DNA methylation (DNAm) influences gene expression and plays a role in establishing a bone cell phenotype [195]. DNAm is involved in the regulation of osteogenic differentiation of mesenchymal cells and the epigenetic mechanisms are important for osteoclast differentiation [196,197]. Responses to stressors, including psychological, behavioural, the hypothalamic–pituitary–adrenal (HPA) axis, and immunological responses, strongly influence DNAm. A heightened inflammatory state lays the foundation for increased vulnerability to tissue damage or fractures. The proinflammatory cytokines, TNFa), IL-1 and IL-6, are interrelated and implicated in changes in bone physiology [198]. When the bone mass is reduced and the microstructure of bone tissue is impaired, it is characterised by fragility and increased fracture susceptibility. Mechanical stress stimulates the skeleton, promotes bone gain, and suppresses bone loss, resulting in improved bone strength and fracture resistance. Osteocytes and osteoblasts coordinate an appropriate response to mechanical loading resulting in localised net bone gain or loss depending on the type of load experienced at specific sites. miRNAs play a critical role in many physiological processes. Altered levels of 134 miRNAs were detected in the plasma from four patients with trochanteric fractures compared with four healthy controls [199]. Li et al. found high miR-214-5p expression in the plasma of patients with hand or intra-articular calcaneal fractures and demonstrated the importance of miR-214-5p downregulation, which resulted in the enhancement of osteoblastic cell viability and resistance to apoptosis [200]. N-methyladenosine (m6A) modification affects cell proliferation, differentiation, and apoptosis in bone-related cells, such as bone marrow mesenchymal stem cells (BMSC), osteoblasts, and osteoclasts. On a molecular level, the epigenetic regulation of m6A affects mRNA processing, nuclear export, translation, and splicing. m6A regulates gene expression (such as ALP, Runx2, Osterix, and VEGF) and signalling pathways (e.g., PTH/Pth1r, PI3K-Akt, and Wnt/β-catenin) [201]. m6A is a methylated adenosine nucleotide that functions through its interaction with the proteins called “writers” (which transfer a methyl group to the N-6 position of adenosine, such as Wilms tumour 1-associated protein (WTAP), methyltransferase-like 3 (METTL3), and methyltransferase-like14 (METTL14), “readers” (which modulate the stability and translation of m6A-modified RNAs, such as YTH family, heterogeneous nuclear ribonucleoprotein (HNRNP) family proteins, insulin-like growth factor 2 mRNA-binding proteins (IGF2BP), leucine-rich pentatricopeptide repeat-containing (LRPPRC), and fragile X mental retardation 1 (FMR1), as well as “erasers”; demethylases that can remove the methyl group of m6A off RNAs, such as fat mass and obesity-associated protein (FTO) and alkB homolog 5 (ALKBH5) [201]. The genome-wide methylation analysis of MSCs from osteoporotic patients with hip fractures revealed significant differences in methylation patterns that were predominantly located in enhancer regions distant from the promoter and gene body. Genes with hypomethylated enhancers and upregulated expression belong to critical fracture healing pathways, including MSC proliferation, osteoblast differentiation. and bone mineralisation [202]. Using the murine mid-diaphyseal fracture model, Hadjiagyrou et al. discovered that target genes of downregulated miRNAs were involved in bone and skeletal development, as well as ossification; target genes of miRNAs that negatively regulated bone remodelling and resorption were upregulated (Table 4) [203].

In standard healing fractures, on day 14, there were five highly expressed miRNAs: miR-140-3p, miR-140-5p, miR-181a-5p, miR-181d-5p, and miR-451 were identified [158]. The early inflammatory phase of fracture healing (days 0–4) coincided with elevated levels of cyclooxygenase (COX)-1, COX-2, lipid mediators, and inflammatory cytokines, and decreased levels of 5-lipoxygenase lipid mediators [204]. It is also well-established that age is a significant risk factor for delayed fracture healing and changes in miRNA expression (miR-21-5p, miR-23a-3p, miR-24-3p, miR-25-3p, miR-27a-3p, miR- 29, miR-31, miR-100-5p, miR-122a-5p, miR-124-3p, miR-125b-5p, miR-148a-3p, and miR-223-3p, miR-3679-3p, and miR-4274) may contribute to this effect [159,160,161]. Bone ageing is characterised by massive loss of bone mass and accumulation of bone marrow adipose tissue (BMAT), which is related to the exhaustion and abnormal differentiation of stem cells [205]. MSCs commonly found in the bone marrow and the spongy bone play important roles in maintaining the dynamic balance of resorption and osteogenesis. With age, the ability of the osteoblasts to differentiate and proliferate decreases while the ability of fat cells increases. This promotes the accumulation of fat in the bone marrow cavity, which negatively affects osteoblasts by reducing their abundance [206].

### 5.4. Epigenetic Mechanisms of Bone Regeneration

Fracture healing is a proliferative physiological process, whereby the body facilitates bone fracture repairs (Figure 2) [207]. Platelets release TGF-β and PDGF for the stimulation and chemotaxis of MSCs and macrophages, which are recruited to the fracture site [133]. Macrophages express fibroblast growth factors 1 and 2 (FGF-1 and FGF-2), IL-1, and TGF-β and promote angiogenesis within the fracture. MSCs differentiate into fibrocytes, chondrocytes, or osteoblasts. Osteoblasts express TGF-β, FGF-1, FGF-2, insulin-like growth factor-I (IGF-I), and BMPs while proliferating chondrocytes express TGF-β, IGF-I, IGF-II, BMP-2, BMP-4, and BMP-7 in association with increased collagen and cartilage matrix synthesis. ECM and hard callus are generated alongside angiogenesis and revascularisation [133,208]. Disturbed vascularisation causes hypoxia in the fracture zone during the initial phase of the fracture healing cascade. miRNAs have been specifically researched in the context of hypoxia and fracture healing. Seven miRNAs have been shown to increase bone regeneration through pathways regulated by hypoxia [161,209]. Adequate blood supply to the fracture is essential for proper bone healing. Various stimuli, such as hypoxia, tissue damage, nutrient demand, and growth and cell proliferation, can trigger angiogenesis [209].

**Table 4:** Roles of miRNA in bone injury.

Type of miRNA	Functional Activity	Reference
miR-1, miR-21, miR-135, miR-155, miR-199a, miR-429, miR-675	Hypoxia-regulated	[161,209]
miR-26a, miR-126, miR-143	Pro-angiogenic	[209,210,211]
miR-22, miR-342	Pro-apoptotic	[161,209,210,211]
miR-21	Inhibit apoptosis	[209,210]
miR-21, miR-31a	Bone resorption	[201]
miR-128, miR-155, miR-182, miR-222	Inhibit bone formation	[209,210]
miR-21, miR-31a	Osteoclast activity	[201]
miR-22	Suppresses osteoblast viability	[161,209,210,211]
miR-21, miR-31a, miR-34c, miR-99, miR-125a, miR-128, miR-142, miR-182, miR-183, miR-218, miR-483	Osteoclastogenesis	[161,201,209,210,211,212]
miR-21, miR-29a, miR-126, miR-128, miR-135b, miR-142, miR-150, miR-218, miR-223, miR-296, miR-451, miR-503	Increase mineralization	[161,209,210,211]
miR-10, miR-17, miR-29b, miR-30c, miR-99, miR-124, miR-125b, miR-133a, miR-138, miR-141, miR-148a, miR-186, miR-193a, miR-200a, miR-203, miR-205, miR-214, miR-320a, miR-320b, miR-409, miR-532, miR-542	Decrease mineralization	[161,200,209,210,211]
miR-9, miR-15b, miR-21, miR-23b, miR-27a, miR-98, miR-128, miR-135b, miR-140, miR-143, miR-149, miR-187, miR-194, miR-409, miR-503, miR-664a, miR-877	Enhance osteogenic differentiation	[161,209,210,211]
miR-29a, miR-194, miR-219a, miR-223, miR-296, miR-302a, miR-5106	Enhance osteoblastic differentiation	[161,209,210,211]
miR-146a, miR-346a	Enhance osteogenesis	[161,209,210,211]
miR-451	Enhances osteoblastogenesis	[161,209,210,211]
miR-10, miR-17, miR-23, miR-31, miR-34a, miR-34c, miE-103, miR-124, miR-125b, miR-133a, miR-138, miR-139, miR-141, miR-145, miR-150, miR-153, miR-181a, miR-186, miR-200a, miR-203, miR-205, miR-206, miR-214, miR-217, miR-320a, miR-320b, miR-342, miR-363, miR-375, miR-383, miR-449b, miR-505, miR-532, miR-765	Inhibit osteogenic differentiation	[161,209,210,211]
miR-22, miR-144, miR-182, miR-193a, miR-542	Suppress osteoblastic differentiation	[161,209,210,211]
miR-29a, miR-140, miR-181a, miR-218, miR-222, miR-335, miR-337	Chondrogenesis	[161,209,210,211]
miR-1, miR-26b, miR-125b, miR-146a, miR-206, miR-214	Inhibit chondrogenic differentiation	[161,209,210,211]

In the process of bone healing, miR-26a, miR-126, and miR-143 are pro-angiogenic. Moreover, miRNAs are involved in the process of bone resorption and regeneration, both after bone fractures, as well as in normal bone remodelling [209,210,211]. miR-21 and miR-31a have been shown to promote osteoclast activity, bone resorption, and osteoclastogenesis [201] in contrast to miR-100, which inhibits the above processes. Osteoclastogenesis is also influenced by several miRNAs (Table 4) [161,209,210,211,212]. In addition, miRNA increases and decreases mineralisation [161,209,210,211,212]. In turn, miRNAs enhance osteogenic differentiation and osteogenesis, i.e., miR-451 enhances osteoblastogenesis and mineralisation, miR-222 enhances the formation of bone and chondrogenesis as well as osteoblastic differentiation, and miR-143 is involved in angiogenesis [161,209,210,211,212]. In addition, miR-124 reduces osteoclast motility. It has also been shown that some miRNAs suppress mineralisation and osteogenic differentiation, i.e., miR-22 has been found to enhance apoptosis and suppress osteoblastic differentiation and osteoblast viability [161,209,210,211,212]. Other miRNAs inhibit osteogenic differentiation and bone formation (Table 4). miR-342 reduces viability, proliferation, and osteogenic differentiation, whilst enhancing apoptosis. miR-193a and miR-542 suppress osteoblastic differentiation and mineralization. miR-144 and miR-182 interfere with osteoblastic differentiation by causing cell cycle arrest. miR-188 promotes adipogenic differentiation. miR-363 reduces osteogenic differentiation and increases cellular senescence [161,209,210,211,212]. Osteocytes act as mechanoreceptors and thereby steer bone formation by direct cellular communication with osteoblasts and osteoclasts [213]. miR-21 prevents osteocyte apoptosis. In turn, miR-23a, miR-24, and miR-27a have been shown to promote osteocyte differentiation [161,209,210]. Osteocytes have also been shown to inhibit osteoblast maturation via miR-29b. Chondrogenesis plays an important role in fracture healing by the formation of cartilage at the early stages of endochondral ossification [214]. Chondrogenesis was shown to be promoted by several miRNAs (Table 4). However, some miRNAs have been shown to inhibit chondrogenic differentiation [161,209,210,211,212]. An increasing number of studies have shown that DNA methylation can regulate the differentiation and apoptosis of osteoblasts and osteoclasts play an important role in the pathomechanism of osteoporosis [211]. Zhou et al. found that 5-AzaC demethylates the genome, increases the expression of osteogenic-related genes, and effectively promotes osteogenic differentiation [215]. Moreover, 5-Aza-dC can demethylate distal-less homeobox 5 (DLX5) and osterix (OSX) gene promoters and upregulate the expressions of osteogenic markers, such as alkaline phosphatase (ALP) and osteocalcin (OCN) [216]. A methylation inhibitor, Hcys, promotes osteoblast differentiation. After Hcys intervention, the expression of the lysyl oxidase (LOX) gene promoter CpG island significantly increased, which inhibited LOX expression, interfered with the formation of bone matrix and ultimately affected the differentiation of osteoblasts [217]. In turn, DNMT3a was found to promote osteoclast differentiation and bone absorption by inhibiting interferon regulatory factor 8 (IRF8), which negatively regulates osteoclast differentiation [211]. Runt-related transcription factor 2 (RUNX2) and OSX are specific transcription factors necessary for bone formation and osteoblast differentiation. During the osteoblast differentiation of MSCs, the level of RUNX2 methylation is decreased so it is thought that RUNX2 methylation plays an important regulatory role in osteoblast differentiation [218]. Hypermethylation of the BMP2 promoter in osteoblasts can inhibit the expressions of bone formation-related genes [211]. Alkaline phosphatase and osteocalcin are secreted mainly by osteoblasts, and both are used as the most common bone formation markers to assess osteogenic activity. Licini et al. found that ALP promoter regions in human osteocytes and osteoblasts had opposite DNA methylation profiles, as those in osteoblasts were hypomethylated, while those in osteocytes were hypermethylated [219]. Most histone modifications are regulated by modifying enzymes that can promote and reverse these specific modifications [211]. In vitro experiments have shown that blocking class I and class II HDACs at the same time or blocking class I HDACs alone can promote osteoblast maturation, bone mineralisation, and the expression of genes related to osteoblast differentiation and maturation, such as type I collagen, osteopontin (OPN), OCN, ALP, OSX, and RUNX2 [220]. HDAC activity plays an important regulatory role in osteogenic differentiation. Osteocalcin is a bone tissue-specific protein that can determine the differentiation and activity of osteoblasts. HDAC3 can inhibit the activation of the OCN promoter by interacting with RUNX2. When OCN transcription is active, histones H3 and H4 of the OCN promoter are acetylated, while histones H3 and H4 are acetylated at low levels when OCN transcription is inactive [211]. In addition, TGF-β can interact with RUNX2 through HDACA4 and HDACA5, which affect histone H4 acetylation in the OCN promoter region [211]. Sirtuin 1 is highly homologous to the silence and yeast information adjustment factor 2 (Sir2) protein, which is a class III HDAC1. SIRT1 can promote the differentiation of osteoblasts (SIRT1/NF-κB pathway), inhibit the formation of osteoclasts (by NF-κB activator ligand, deacetylation of FoxO, and reduced ROS), regulate bone reconstruction, and affect bone metabolism [221]. Methyltransferases and demethylases regulate the expressions of related genes in osteoblasts and osteoclasts. Inhibitors of the differentiation of MSCs into osteoblasts include the enhancer of zeste homolog 2 (EZH2). In turn, lysine-specific demethylase 1 (LSD1) and jumonji domain-containing protein (JMJD) enhance bone formation (by BMP2 and WNT7B), promote osteoblast differentiation (via Runx2, OSX, and OCN), and promote the differentiation of osteoclasts (RANKL) [211,222]. In addition, 51 lncRNAs were found to be abnormally expressed in a study of postmenopausal women with osteoporosis (e.g., H19, LncRNA p21, DNACR, BDNF-AS, Bmncr) [223].

## 6. Traumatic Brain Injury (TBI)

### 6.1. Pathophysiology of Trauma

**Figure 3:**
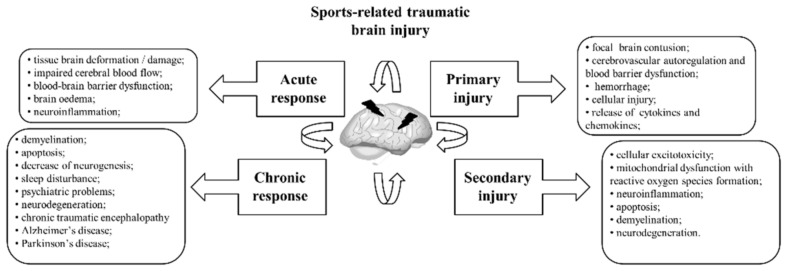
Molecular characteristic of distinct phases of sports-related traumatic brain injuries (primary and secondary) with short- (acute) and long-term (chronic) effects.

Sports, after car accidents, is the second most important cause of traumatic brain injury [224]. TBI occurs when a force transmitted to the head causes impairment of brain functioning and neuropathologic damage. The incidence of TBI is increasing globally. Approximately 1.6 million to 3.8 million sports- and recreation-related TBIs are estimated to occur in the United States annually [225]. Sports-related TBIs are believed to account for up to 20% of all TBIs with half of these occurring in children and adolescents [226]. Figure 3 presents the characteristics of sports-related TBI.

A TBI is classified in terms of the type and severity of the injury. Regarding the type, classification can be based on the presence of focal lesions in focal and diffuse injuries. The first one includes concussion intraparenchymal haemorrhage, subdural, or epidural hematoma, and is usually caused by direct physical contact. Diffuse injury is triggered by acceleration and deceleration of the brain. It accounts for approximately 70% of TBI cases and includes axonal, hypoxia-ischaemia, and microvascular injuries affecting various anatomical regions during a single event [227]. Other classifications include primary (acute) and secondary (delayed) injuries (Figure 3) [228].

Primary injuries are directly caused by external mechanical forces, such as acceleration and deceleration linear forces, rotational forces, associated with blast injuries, and damage to whole neurons, their axons, dendrites, glial cells, and blood vessels. This state can initiate delayed and prolonged secondary damage that spreads via multiple molecular mechanisms. Several factors contribute to the induction of secondary injuries, such as hypoxia, excitotoxicity, mitochondrial dysfunction, oxidative stress, neuroinflammation, axon degeneration, and apoptosis [224,227,229].

To assess the severity of TBI, the Glasgow coma scale (GCS), post-concussion symptom scale (PCSS), loss of consciousness (LOC), and duration of post-traumatic amnesia (PTA) indicators are applied [230]. Upon clinical examination, according to the GCS, TBI is most commonly subdivided into mild, moderate, and severe [231]. Diagnosis based on the GCS ranks functional ability from 1 (worst outcome) to 15 (best outcome), with a mild injury defined between 13 and 15. It provides an assessment of the patient’s condition based on three criteria: eye-opening and closing reactions, motor reactions, and verbal communication. Mild TBI caused by either a direct blow to the head, face, neck or elsewhere on the body with an “impulsive” force transmitted to the head is most common in sports. This involves a closed head injury and includes concussion, contusion, diffuse axonal injury, and intracranial hematoma (epidural hematoma, subdural hematoma, subarachnoid haemorrhage, and intra-parenchymal haemorrhage) [231].

**Figure 4:**
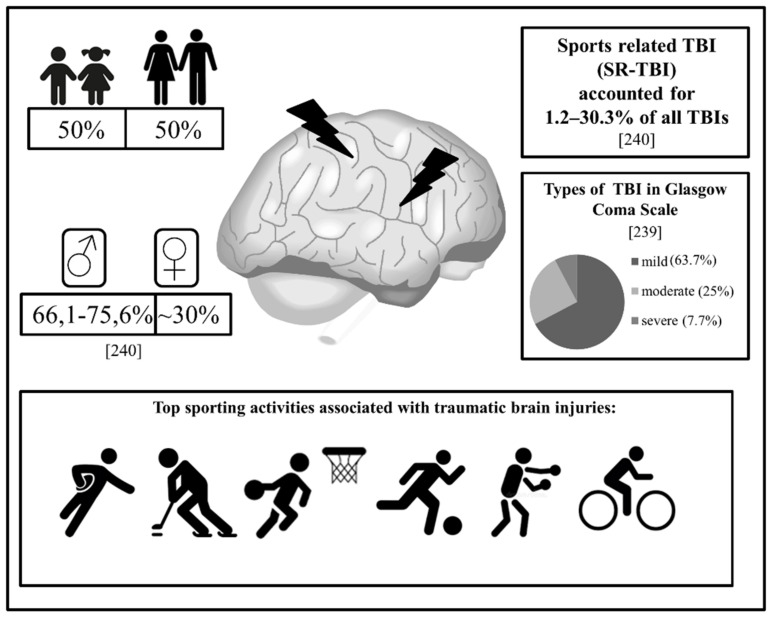
Summary of sports-related TBI causes and prevalence. Figure data obtained from [239,240].

In sports, TBI is also characterised by the six-point PCS [229,232]. This assessment takes into account various physical, sleep-related, emotional, and attentive symptoms. PCSS classifies concussions into no symptoms (score of 0), mild (from 1–2), moderate (from 3–4), and severe (5–6) categories. Moreover, the LOC and PAT indices are used to assess the severity of TBI. The LOC divides TBI into three categories: mild (LOC < 30 min.), moderate (LOC > 30 min to 6 h), and severe (LOC > 6 h) TBI. The PAT measures the time elapsed from the injury to the return of orientation or continuous memory. Based on this assessment, TBI is classified as mild (PAT < 2 h), moderate (PAT < 1 week), or severe (PAT > 1 week) [227,232]. The data suggest that the different types and severities of sports-related TBI have unique short and long-term outcomes and, thus, may represent different types of diseases [233]. For example, mild traumatic brain injury significantly increased the risk of developing Alzheimer’s disease (AD), Parkinson’s disease (PD), and chronic traumatic encephalopathy (CTE) in former professional athletes (Figure 4). Moreover, these injuries interfered with lifestyles through physical, emotional, and psychosocial changes that ultimately affected daily activities [234,235]. Studies have shown that TBI is a complex disorder in which several pathophysiological processes may occur depending on the injury subtype, including axonal injury, astrogliosis, and neuronal injury or death [228]. Mild traumatic brain injury (or concussion) is a common consequence of a collision, fall, or another form of contact in sports [236]. Concussion may be complicated by cerebral oedema related to second impact syndrome, cumulative neuropsychologic deficits, intracranial bleeding, or post-concussion syndrome. At the beginning (primary injury), when the brain is subjected to rapid acceleration, deceleration, and rotational forces, it elongates and deforms, which stretches individual cellular components such as neurons, glial cells, and blood vessels, and alters membrane permeability [229]. The cellular injury does not uniformly affect all axonal populations. Studies have shown that smaller, unmyelinated axons may be more susceptible to damage from concussive forces than larger myelinated axons. This is important, especially in the case of children who play sports, and who are exposed to concussions [228]. Immediately after a biomechanical injury to the brain, there is a “neurometabolic cascade of concussion”. The rapid cellular membrane stretch can result in an unregulated flux of ions. Calcium dysregulation associated with traumatic brain injury has several effects [228,237].

It is known that secondary damage is also linked to neuroinflammation [237]. It begins with the complement and microglial activation. Following TBI, there is an immediate disintegration of the blood–brain barrier (BBB) due to the mechanical forces imposed on the brain tissue. Endothelial cells, components of the BBB, lose their tight junctions. Complement enhances the influx of peripheral leukocytes, which penetrate the weakened BBB. Increased BBB permeability results in the efflux of plasma proteins, which further increases the inflammatory response. Microglial activation causes the release of cytokines and chemokines (i.e., TNFα, IL-1β, IL-6) that affect the permeability of the BBB. Interestingly, microglia have two activation phenotypes (M1 and M2), which depend on their interactions with pro- or anti-inflammatory mediators.

Neuroinflammation also leads to the accumulation of ROS, which are responsible for lipid peroxidation, protein carbonylation, and DNA oxidation. Finally, the activation of caspases promotes apoptosis, necrosis, and autophagic mechanisms [224]. These processes require large increases in glucose metabolism [228]. This post-concussive hypermetabolism occurs due to insufficient cerebral circulation, with a widening disparity between glucose supply and demand, producing a cellular energy crisis.

### 6.2. Molecular and Genetic Basis of TBI

It is also worth mentioning that numerous genes have been implicated in pathophysiology and outcomes following moderate to severe TBI. More recently, considerable attention has focused on genes associated with mild and repetitive TBI among professional athletes. Apolipoprotein E (*APOE*) is the most studied gene with respect to outcomes after mild TBI. It has been reported that boxers with the *APOE4* allele have suffered from chronic traumatic encephalopathy (CTE). Moreover, it has been observed that older professional football players with *APOE4* allele scored lower on cognitive tests than those without [238,239,240]. More recently, in a literature review of 47 cases of neuropathologically verified CTEs, 46 of which were athletes, APOE genotyping was reported in 10 cases, and of these, half carried at least one *APOE4* allele [241]. Another study focused on the tau protein, a member of a large family of microtubule (MT)-associated binding proteins, which stabilises the assembly of MT in neurites and is abundantly expressed in the axons of neurons in both the CNS and in the peripheral nervous system (PNS), and to lower levels in oligodendrocytes and astrocytes. Previous studies among amateur boxers and professional hockey players have found elevated levels of tau in the cerebrospinal fluid and plasma. This change significantly correlated with the number and severity of head injuries. Unfortunately, few studies have evaluated the association between tau genetic polymorphisms and TBI. In fact, to date, only one group has reported on tau polymorphisms and acute head injury. Terrell et al. performed tau genetic testing (Ser53Pro and His47Tyr in exon 6) on 195 college football and soccer athletes and observed the TT genotype of tau Ser53Pro (rs10445337) to be weakly associated with an increased risk of concussion [242]. More recently, the same analysis was repeated in a larger cohort of college athletes (*n* = 3218), and no significant association was observed between either of the tau SNPs and concussion incidence [241]. Other genes involved in the pathophysiology of TBIs are related to the inflammatory response (pro-and anti-inflammatory cytokines), such as IL-1. A number of genetic polymorphisms have been identified in IL-1 that influence gene expression and are associated with brain injury [241]. For example, the presence of the IL-1B +3953 T and −511 G alleles are associated with unfavourable outcomes. IL-1RN*2 is associated with an increased likelihood of cerebral haemorrhage after TBI. In the case of IL-6, in a recent prospective study of 3255 college athletes, Terrell et al. observed a significant association between the IL-6 −572 C/C genotype and concussion risk [242]. Few clinical studies have examined TNFα polymorphisms in the context of TBI. It has been reported that only TNFα −308 A (allele 2) carriers show unfavourable outcomes compared to non-carriers. Taken together, changes in one allele may play a significant role in prognosis and recovery after TBI. However, it should be remembered that recovery is polygenic in nature. A number of gene interactions and multiple molecular pathways are involved in this process.

### 6.3. Epigenetics of TBI

On the other hand, one must also consider the roles of epigenetic mechanisms in sports-related brain injuries as processes that can affect gene expression without altering the DNA sequences [241]. TBI research is problematic in athletes. The current approach mostly involves observational trials and post-mortem analysis. Due to the ethical implications and methodological issues associated with studies on human subjects, the vast majority of TBI research is conducted using animal models (weight drop, fluid percussion, controlled cortical impacts) [243]. In these studies, it is easier to assess the involvement of epigenetic mechanisms, such as DNA methylation, histone modification, and non-coding RNAs regulation of gene expression in the post-injured brain. Although the field of epigenetics is now well-established, interest in the epigenetic mechanisms involved in TBI pathophysiology has only recently gained some traction. Potential changes, for example, in methylation status following TBI, remain unclear, but these changes might influence massive changes in gene expression participating in recovery and repair processes [244]. As mentioned above, microglial activation is an important step in the pathophysiology of TBI [227]. The activation of this cell population contributes to post-injury inflammation by the upregulation of certain membrane proteins and the expressions of various cytokines, which take part in regeneration but are also involved in deleterious changes in the central nervous tissue. In this regard, epigenetic changes seem to be highly important in the coordination of inflammatory gene expression, repair, and recovery processes in central nervous system lesions [244]. Zhang, using the rat weight-drop model of TBI, reported global hypomethylation within the first day after injury in regions of widespread necrosis in the somatosensory cortex, and a slightly delayed response (day 2) in the peripheral regions of the lesions. Moreover, deeper analysis with double immunostaining revealed hypomethylation specifically in ED1^+^ or EMAP-II^+^ microglia/macrophages^,^ but not in W3/13^+^ and OX22^+^ cells. These results suggest that injury-induced hypomethylation defines a subpopulation of infiltrating reactive microglia that may exert particular functions during secondary injury [244]. An interesting finding is that dexamethasone caused a reduction in hypomethylated ED1^+^ microglia/macrophages on day 2 after TBI. Importantly the ratio of this subpopulation to total ED^+^ microglia/macrophages remained comparable to the control group. This result suggests that dexamethasone has an inhibitory effect on inflammatory cell infiltration and might play a role in changes to methylation status in this model of TBI [244]. In another study, Schober et al. used a controlled cortical impact model of TBI used in rats [245] and showed that TBI was associated with DNA hypermethylation at one region of the *IGF-1* gene (exon 5 and upstream); DNA hypomethylation was also found at another site (downstream of exon 5) [245]. These epigenetic changes correlated with a higher expression of *IGF-1B* (insulin-like growth factor 1 B), the splice variant of *IGF-1*, and a key, endogenously-produced neuroprotective factor. Most knowledge about DNA methylation following TBI has been gained from animal studies. However, Lee et al. recently quantified global DNA methylation by specifically measuring ratios of 5-methylcytosine (5-mc) in an enzyme-linked immunosorbent assay [246]. Blood samples were obtained from 25 volunteer college students, including 14 healthy controls (64.3% females; mean age of 22.0 years) and 11 mild TBI cases (27.3% females; mean age of 28.7 years) who self-reported TBI history (63.6% multiple; 2.5 ± 1.29 injuries), with 7.1 years on average elapsed following the last injury. Moreover, 36.4% of traumatic cases were sports-related. The peripheral epigenetic marker comparison finding showed a significantly higher blood global methylation ratio (5-mC%) in mTBI cases than in the controls [246]. Hamdeh and co-workers have published a preliminary report that provides evidence of an altered DNA methylome in the injured human brain [247]. In this study, the DNA methylation statuses of genes related to neurodegeneration, such as the amyloid β precursor protein (*APP*), microtubule-associated protein tau (*MAPT*), neurofilament heavy (*NEFH*), neurofilament medium (*NEFM*), and neurofilament light (*NEFL*), were analysed in fresh, surgically resected human brain tissue from 17 severe TBI patients and compared with brain biopsy samples from 19 patients with idiopathic normal pressure hydrocephalus (iNPH). TBI caused alteration of methylation in thirty-eight CpG sites of the *APP*, *MAPT*, *NEFH*, and *NEFL* genes. Among the top 20 differentially methylated CpG sites, 11 were in the *APP* gene [247]. In addition, an epigenome-wide association study (EWAS) evaluating 828,888 CpG sites revealed 308 differentially methylated CpG sites in genes related to cellular/anatomical structure development, cell differentiation, and anatomical morphogenesis [247]. In another study, Liu et al. attempted to identify epigenome-wide DNA methylation patterns associated with cerebral oedema and intracranial hypertension after TBI. DNA was extracted from ventricular cerebrospinal fluid samples at three different post-injury time points from patients with severe TBI (*n* = 89 patients). The authors reported a novel potential relationship between intracranial hypertension after TBI and an acute, non-sustained reduction in DNA methylation at cg22111818 in the repulsive guidance molecule (A-*RGMA*) gene. It is known that RGMA may be involved in the pathogenesis of CNS diseases, such as multiple sclerosis, neuromyelitis optica spectrum diseases, cerebral infarction, spinal cord injury, Parkinson’s disease, and epilepsy [248]. Thus far, much of the discussion has focused on the changes in enzymes controlling DNA methylation, such as DNMTs. Using a rat model, Lundberg et al. investigated the expressions and cellular localisations of DNMTs. It was observed that, in the case of astrocytes, DNMT1 was located in the nucleus and cytoplasm, in contrast to normal neuronal nuclear localisation [249]. Moreover, double staining with DNMT1 and nestin showed co-localisation in some reactive astrocytes in the nucleus alone, while in others, the expression patterns were evident both in the nucleus and cytoplasm, in a brain region-specific manner [249]. In another study, Sagarkar et al. employed the closed-head injury paradigm to induce mild TBI in rats and reported that mTBI altered DNMT function and demethylation factors (GADD45a and GADD45b) in the amygdala. Moreover, TBI induced DNA methylation in the *BDNF* (brain-derived neurotrophic factor) gene promoter [250].

Research has demonstrated that sleep disturbances are common following sports-related TBI [246,251]. The presence of any sleep disturbance following TBI of any severity ranges from 42–70% of cases [252]. It was observed that the expression of genes involved in the sleep cycle regulation, such as *Aanat* (arylalkylamine N acetyltransferase), *Nos1* (nitric oxide synthase 1), *Il1r1* (interleukin-1 receptor type 1), *Homer1* (Homer scaffolding protein 1), *Chrna3* (cholinergic receptor nicotinic α 3 subunits), and *Per3* (period circadian clock 3) decreased in the frontal cortexes of rats as a result of increased DNA methylation [245]. In the last few years, it was found that TBI also promotes histone modifications [245]. In the previously mentioned study, Schober et al. observed that controlled cortical impact increased the occupancy of H3K36me3 at the P1 region of the *IGF-1* gene promoter but decreased at the P2 promoter region [245]. On the other hand, Gao et al., using a controlled cortical impact model of TBI in immature rats, showed a significant decrease in histone H3 acetylation in the hippocampal CA3 region at 6 and 24 h post-injury, along with a decrease in histone H3 methylation at 6, 24, and 72 h after injury [249]. Similar results were obtained by Zhang et al. in a fluid percussion model in rats and by Shein et al. in a weight-drop mice model of TBI [244,253]. In that context, the contribution of HDAC inhibitors was interesting, especially in terms of normalising the deleterious consequences of TBI. Several studies in the last few years have shown that post-injury administrations of classes I and II HDAC inhibitors, such as ITF2357, scriptaid, and CI-994, increase the histone H3 and H4 acetylation levels, increase the expressions of neurotrophic factors, and promote neuronal rewiring and functional recovery following TBI [253,254,255]. For example, administration of ITF2357 in a mouse model 24 h after closed-head injury improved neurobehavioral recovery. This functional benefit was accompanied by decreased neuronal degeneration and reduced lesion volume (22% reduction). Importantly, all events were preceded by increased acetylated histone H3 levels and the attenuation of injury-induced decreases in cytoprotective heat-shock protein 70 kDa and phosphorylated Akt. Moreover, reduced glial accumulation and activation were observed 3 days post-injury, and total p53 levels at the areas of injury and caspase-3 immunoreactivity within microglia/macrophages in the trauma areas were elevated [253]. Using a controlled cortical impact (CCI) model of TBI in mice, Wang et al. demonstrated that scriptaid protects white matter up to 35 days after TBI, by reducing abnormally dephosphorylated neurofilament proteins, increasing the myelin basic protein, accompanied by the anatomic preservation of myelinated axons, and improving nerve conductions [254]. Furthermore, scriptaid shifted microglia/macrophage polarisation toward the protective M2 phenotype and mitigated inflammation. In primary cocultures of microglia and oligodendrocytes, this HDAC inhibitor increased the expression of microglial glycogen synthase kinase 3 β (*GSK3β*), which phosphorylates and inactivates phosphatase and tensin homologue (PTEN), thereby enhancing phosphatidylinositide 3-kinases (PI3K)/Akt signalling and polarizing microglia toward M2 [254]. It has been suggested that an increase in GSK3β in microglia (and their phenotypic switch to M2) is associated with increased preservation of neighbouring oligodendrocytes [254]. In a recent study, Sada et al. reported that administration of a class I HDAC inhibitor in the CCI mouse model of TBI increased the number of synaptic buttons in rewiring corticospinal fibres and improved the recovery of motor functions [256]. Immunohistochemistry results showed that HDAC2 was mainly expressed in the neurons of the mouse spinal cord under normal conditions. After TBI, HDAC2 expression was increased in the spinal cord after 35 days, whereas BDNF expression decreased after 42 days [256]. Administration of CI-994 (N-acetyldinaline) increased BDNF expression after TBI. Knockdown of HDAC2 elevated H4K5ac enrichment at the BDNF promoter, which was decreased following TBI. Moreover, it was shown that HDAC inhibitors decreased the permeability of the BBB (blood–brain barrier), reduced the degree of neural damage, and improved cognitive and functional outcomes after TBI [245]. In recent years, miRNAs have been identified as key regulators in neural functioning in various pathophysiological conditions [257]. miRNAs have been implicated in both the primary and secondary damage responses to TBI. Injured neurons may release miRNAs into the extracellular space, where their small sizes allow them to navigate the BBB (blood–brain barrier), facilitating peripheral sampling. In the cellular response to secondary damage, neurons use miRNA signalling to regulate synaptogenesis and neuroplasticity. As a result, miRNA profiles during the subacute period may also telegraph the trajectory of brain recovery [258]. There are many pathways targeted by miRNAs in the pathophysiology of TBI, including: adrenergic signalling, AMP kinase activity, oestrogen signalling, fatty acid metabolism, GABAergic signalling, synaptic vesicle cycling, and TGF-β signalling [258]. While studies demonstrating DNA methylation and histone changes in response to sports-related brain injuries have mostly used animal models, studies on miRNAs have recently begun to be performed in human athletes. However, different types of miRNAs involved in TBI have been well-documented in rodent models [227].

From a clinical point of view, human miR-21, miR27a, let-7, and miR107 are the most intensively studied miRs in TBI. The expression levels of miR-21 in various cells (neurons, astrocytes, and microglia) of the CNS increase after TBI [259]. The function of miR-21 in neuroinflammation is paradoxical. It directly targets PDCD4, Smad7, and Spry1, and plays an anti-inflammatory role in neurologic diseases. It also has detrimental effects on MS and other inflammatory conditions. The role of miR-21 in neuroinflammation as a consequence of TBI is not well understood. Upregulation of miR-21 reduces neuronal apoptosis after TBI by significantly decreasing the expression level of PTEN, and increasing the phosphorylation of Akt, reducing the downstream apoptosis-related proteins of the PTEN-Akt signalling pathway [259]. The signalling molecules that activate the miR-21 response to astrogliosis may be a distinct set of yet unidentified gene targets (neither PDCD4 nor PTEN). miR-21 regulates the expression of inflammatory cytokines, NF-κB signalling, the expression of apoptosis factors, Akt signalling, and excitation of the Ang-1/Tie-2 axis, thereby preventing BBB damage after TBI. miR-21 promotes angiogenesis after TBI by upregulating VEGF expression and activating the Ang-1/Tie-2 axis [259]. Moreover, miR-27a has been reported to be a brain-specific miRNA that is aberrantly expressed in the brain suffering from TBI [260]. The downregulation of miR-27a and an increase in FoxO3a were observed in the post-TBI hippocampus. Overexpression of miR-27a significantly attenuated neurological deficits and brain injury, especially suppressed autophagic activation after TBI. Furthermore, it was identified that miR-27a directly targeted the FoxO3a 3’UTR region to reduce the FoxO3a protein expression. Knockdown of FoxO3a significantly reversed the high levels of autophagy-related genes induced by TBI [260].

The Lethal-7 (let-7) miRNA is conserved across species (from *Caenorhabditis elegans* to humans) and was the first miRNA to be identified in humans [261]. It has also been shown that caspase-3 is the putative target gene of let-7c-5p. Lv et. al., have reported that let-7c-5p inhibits the expression of caspase-3 [262]. Other studies have shown that, after ischemic stroke, overexpression of let-7 reduces post-stroke neurotoxicity and improves neurologic outcomes, an effect that might be caused by the let-7-mediated reduction of caspase-3. These effects were also associated with reduced microglial activation. It was also demonstrated that inhibition of caspase-3 by let-7c-5p reduced the TBI-induced activation of protein kinase C delta (PKC-δ), which regulates NF-κB activation through IKK complexes [262].

In Table 5, we summarize other human clinical studies, assessing circulating miRNAs after sports-related TBIs that have been investigated during the last few years.

The identification of circulating specific miRNAs in blood or saliva and their targets may help diagnose the spectrum of TBI. Moreover, recent findings suggest that these miRNAs contribute to the process of recovery, enabling natural mechanisms of neuroprotection [267,268,269]. As mentioned at the beginning of this section, mitochondria play a central role in the pathophysiology of TBI. Multiple findings have demonstrated mitochondrial dysfunction as one of the hallmarks of secondary injury after TBI [227]. However, it has also been recently suggested that mitoepigenetic changes could result from traumatic brain injury, a well-known risk factor for neurodegeneration [245]. In response to that, the mitochondrial genome has also received some attention. Recent investigations have revealed impaired methylation levels of the mitochondrial regulatory region (D-loop region) in animal models, post-mortem brain regions, and circulating blood cells of patients with Alzheimer’s disease, Parkinson’s disease, and amyotrophic lateral sclerosis. Those studies also revealed that mtDNA D-loop methylation levels are subjected to dynamic regulation during the progression of the neurodegenerative process and are inversely correlated with the mtDNA copy number. The methylation levels of mtDNA regions other than the D-loop have been poorly investigated in patients with neurodegenerative disorders or animal models, and evidence of impaired methylation levels is often limited to a single study. Moreover, it is known that the mtDNA haplogroups K and T are involved in protective effects in TBI. McDonald et. al. showed that there is a strong relationship between mtDNA deletions in cases of acute brain injury [270]. Unfortunately, so far there have been no studies about mitoepigenetics conducted on athletes.

## 7. Summary and Future Directions

PA offers many benefits for people in all age groups. Both professional athletes and amateurs face contusions and sports-related injuries due to training or sporting accidents. The accumulation of numerous micro and macro changes in tissues exposed to intense physical exertion, as well as genetic factors, not only affect the course of a sports career, but also its length. It is important from the perspective of sports professionals, as well as for those performing regular, amateur sporting activities, to maintain the proper levels of training, without enduring several/frequent injuries. Currently, we understand how significant it is to comprehend bodily reactions and responses on multiple levels, including the physiological and molecular levels. In the past 20 years, epigenetics, as a relatively new area of science, has provided us with a vast amount of data. Specifically, this epigenetic perspective explains environmental effects on the functions of the body. The observation of epigenetic mechanisms, such as DNA methylation, histone protein methylation, and acetylation, as well as miRNA expression, has the potential to become a useful tool in sports medicine, both as a predictor of approaching pathophysiological alterations and as a biomarker of injuries that have already taken place [271]. For example, research into epigenetic changes after brain injury has shown differences in the concentration of miRNAs in plasma, saliva, and cerebrospinal fluid (CSF) at various time points after TBI [264]. This may be related to TBI symptoms and may improve diagnostics and further therapy. It is believed that the discovery of reliable epigenetic markers in blood or saliva will decrease the number of under- or misdiagnosed TBIs. Epigenetic changes, mainly changes in miRNA levels, may become new markers of sporting injuries or treatment targets, which could be useful in sports medicine and traumatology. The possibility of acquiring material during non-invasive procedures, such as blood and saliva to assess miRNA changes, may become a useful diagnostic and prognostic tool to help improve care and decrease the number of underdiagnosed tissue injuries (mainly TBIs). Further studies are needed to confirm the utility of miRNA level estimations for diagnostic and treatment purposes. Moreover, the first experimental research on the exploitation of agonists and antagonists of miRNAs in the modulation of epigenetic changes for therapeutic purposes, i.e., tendon healing and regeneration, are reviewed in a paper by Giordano et al. 2020 [149]. The measurements of circulating miRNAs may be helpful in diagnostics as well as in planning the regeneration time between sporting events (competitions). We hope that a detailed understanding of the epigenetic mechanisms regulating the complex processes of tissue damage and regeneration will allow, in the future, a reduction in the number of injuries in sports and a more rapid return to training.

## Figures and Tables

**Table 1:** miRNAs present in circulating blood and other body fluids.

Type of miRNA	Reference
↑miR-1, ↑miR-126, ↑miR-133a, ↑miR-206, ↑miR-208, ↑miR-146a, ↑miR-221, ↑miR-222, ↑miR-499, mir-223, ↑miR-24, ↑miR-149, ↑miR-30a, ↑miR-125a-5p	[98,99,100,101,102,103,104,105,106,107,108]
↓miR-26a, ↓miR-29b, ↓miR-15b	[100,105]

↑ upregulation; ↓ downregulation.

**Table 2:** miRNAs featured in tendons and bone injuries.

Type of miRNA	Results and Main Conclusion	Reference
miR-29b	Deterioration of tendon adhesion;	[147,148,149]
miR-28, miR-17-92	mediate oxidative stress and induce tenocyte apoptosis;	
miR-135a	regulates TSPC senescence by targeting ROCK1;	
miR-608, miR-499	related to pathogenesis of tendinopathy; regulate MYB and CUGBP2;	
miR-210	regulates angiogenesis;	
miR-378, miR-133, miR-206, miR-140, let-7a, let-7e, miR-338, miR-381, miR-743	related to mechanical stimuli	
miR-421-5p	Regulates the local expression of MMP2, MMP9, and drives angiogenesis by increasing VEGF	[150]
miR-30a-5p, miR-140-3p, miR-210-3p, miR-222-3p, miR-324-3p, miR-425-5p	Deregulated in patients in chronic tendinopathy;	[151,152]
miR-29c	progressive repression depending on the severity of the tendon pathology	[152]
miR-324	Inhibits the expressions of MMP-2 and MMP-9 and might promote tendon disorganization	
miR-140-3p	Negatively regulates nuclear factor-κB (NF-κB) inflammatory signalling.	[153]
hsa-miR-145-5p, hsa-miR-99a-5p, hsa-miR-100-5p, hsa-miR-150-5p, hsa-miR-193b-3p, hsa-miR-103a-3p, hsa-miR-31-5p, hsa-miR-195-5p, hsa-miR-497-5p, hsa-miR-15a-5p, hsa-miR-16-5p, hsa-let-7b-5p	Downregulate miRNA associated with 216 genes in rotator cuff tendon injury	[154]
hsa-miR-297	Upregulates miRNA associated with 216 genes in rotator cuff tendon injury	
miR-148a-3p	Promotes thrombospondin-4 expression and enhances angiogenesis during tendinopathy;	[155]
miR-124	suppresses collagen formation of human tendon	
miR-124-3p	Directly binds and suppresses the expression of EGR1 and suppresses the synthesis of collagen during tenogenic differentiation	[149]
miR-30b-5p	Involved in cartilage degradation in rats and regulates chondrocyte apoptosis and migration by targeting Hoxa1	[156]
mir-500, miR-548j	Regulates the processes associated with matrix remodelling	[157]
miR-214-5p	Regulates osteoblastic cell viability and apoptosis	[156]
miR-140–3p, miR-140-5p, miR-181a-5p, miR-181d-5p, miR-451	Highly expressed miRNAs in standard healing fractures on day 14	[158]
miR-21-5p, miR-23a-3p, miR-24-3p, miR-25-3p, miR-27a-3p, miR-29, miR-31, miR-100-5p, miR-122a-5p, miR-124-3p, miR-125b-5p, miR-148a-3p, miR-223-3p, miR-3679-3p, miR-4274	Associated with the development of osteoporosis and bone fracture risk	[159,160,161]

**Table 3:** Roles of miRNA in tendon injury.

Type of miRNA	Functional Activity	Reference
miR-1, miR-21, miR-28-5p, miR-34 family, miR-100, miR-133a, miR-133b, miR-205, miR-221, miR-222, miR-337-3p, miR-378	Tendon homeostasis	[147,171]
miR-34 family, miR-199 family, miR-205-5p, miR-499	Proliferation	[171,172]
miR-21-5p, miR-21a-3p, miR-29b, miR-34 family	Tendon adhesion	[147,171]
miR-21-5p, miR-34 family, miR-125a-5p, miR-145-5p, miR-151a-3p, miR-199a-5p, miR-382-5p, miR-498	Tendon ECM	[147,171]
miR-17-92, miR-28, miR-34 family, miR-181 family	Apoptosis	[150,171,172]
miR-199 family	Cell survival	[172]

**Table 5:** Clinical studies of circulatory miRNAs after sports-related TBIs.

Biological Material Analysed	Examined Group	Type of miRNA	Results and Main Conclusion	Ref.
saliva	A total of 6 rugby professional and semi-professional athletes and 6 controls	miR-27b-3p miR-142-3p miR-let-7i-5p miR-107 miR-135b-5p	Expression was significantly upregulated in concussed athletes; univariate ROC curve analysis showed that the differentially expressed miRNAs could be considered good classifiers of concussion.	[263]
serum	A total of 27 collegiate athletes after sports-related concussion (~41% ♂, ~75% ♀ white, age 18.8 ± 0.8 years)	miR-153-3p miR-223-3p miR-26a-5p miR-423-3p miR-let-7a-5p	Significant increase in expression following SRC for miR153-3p (59% of the participants increased post-SRC), miR223-3p (70% increased), miR-let-7a-5p (65% increased); no statistically significant associations between changes in miRNA expression and clinical test scores, acute symptom severity, or clinical recovery time.	[264]
plasma	A total of 28 amateur Australian rules football players after sports-related concussions (20 ♂ and 8 ♀) and the control group, 99 Australian rules; football players (62 ♂, 37 ♀)	miR-19b-1-5p miR-20b-5p miR-21-5p miR-27a-3p miR-28-5p miR-103a-3p miR-106a-5p miR-125-5p miR-142-3p miR-194-5p miR-221-3p miR-223-3p miR-301b miR-338-5p miR-643 miR-769-5p miR-1260a miR-1290	miR-27a and miR-221 were decreased in the sub-acute stages after SRC; plasma levels of these miRNAs were inversely correlated with SRC symptom severity.	[265]
Serum	A total of 53♂ (30 non-athlete control subjects and 23 collegiate student football athletes)	miR-20a miR-505* miR-362-3p miR-30d miR-92a miR-486 miR-195 miR-9-3p miR-151-5p	In athletes with declining neurocognitive functioning over the season, concentrations of miRNAs increased. Significant negative correlations with miR-505*, miR-30d, miR-92; miRNAs correlating with balance problems: miR-505*, miR-30d, miR-151-5p; correlating with poor reaction times: miR-20a, miR-505*, miR-30d, miR-92, and miR-151-5p.	[266]
Serum	Professional soccer players (44 after accidental head impact and 68 after repetitive headers), controls—from a bank of serum (young healthy individuals)	miR-1-3p miR-7-5p miR-16-p miR-17-5p miR-18a-5p miR-20a-5p miR-24-3p miR-27a-3p miR-93-5p miR-106b-5p miR-107 miR-130b-3p miR-122-5p mi-143-3p miR-150-5p miR-204-5p miR-206 miR-499a-5p miR-885-5p let-7c-5p	Dysregulation of expression depends on the type of injury (accidental head impacts or repetitive headers) and time (1 or 12 h after injury)	[267]
saliva	A total of 310 individuals: with no history of concussion (*n* = 230), single concussion (*n* = 56), recurrent concussion (*n* = 24)	20 miRNAs	miR-28-3p and miR-339-3p demonstrated relationships with the number of prior concussions	[258]

♀ female; ♂ male.

## Data Availability

Not applicable.
